# Inclusive mobile brain-body imaging achieves equivalent EEG data quality across racial groups

**DOI:** 10.1371/journal.pdig.0001638

**Published:** 2026-08-07

**Authors:** Sodiq Fakorede, Ke Liao, KathleenMae Rogers, Kai Cheng, Lydia Pemberton, Laura E. Martin, Hannes Devos

**Affiliations:** 1 Department of Physical Therapy, Rehabilitation Science, and Athletic Training, University of Kansas Medical Center, Kansas City, Kansas, United States of America; 2 Hoglund Biomedical Imaging Center, University of Kansas Medical Center, Kansas City, Kansas, United States of America; 3 Department of Population Health, University of Kansas Medical Center, Kansas City, Kansas, United States of America; 4 Mobility Core, University of Kansas Center for Community Access, Rehabilitation Research, Education and Service, Kansas City, Kansas, United States of America; 5 University of Kansas Alzheimer’s Disease Research Center, Kansas City, Kansas, United States of America; 6 Landon Center on Aging, University of Kansas Medical Center, Kansas City, Kansas, United States of America; Harvard University, UNITED STATES OF AMERICA

## Abstract

Electroencephalography (EEG) research systematically excludes participants with textured hair, limiting generalizability. While inclusive hardware offers a solution, it remains unvalidated in dynamic settings. This study bridges this ecological gap by determining if equitable data quality is achievable across racial groups during a complex Mobile Brain/Body Imaging (MoBI) paradigm. We recruited 17 older adults from racially and ethnically underrepresented groups (REUG) and 17 age-and-sex-matched White older adults. Participants completed an auditory oddball task while sitting and during active standing. EEG was recorded using a dry-brush-electrode system paired with culturally sensitive procedures. The primary outcome was event-related potential (ERP) data quality, quantified using the Standardized Measurement Error (SME) for P3 amplitude and latency. Total data loss was comparable between White (8.29%) and REUG participants (9.88%), with no group differences (p = 0.91) or group×condition interactions (p = 0.82). We found no significant main effects of group or group-by-condition interactions on any SME measure (all p > 0.05), and equivalence testing confirmed that SME for P3 amplitude and latency was statistically equivalent in 16 of 18 stimulus × postural comparisons. A sensitivity analysis restricting the REUG group to participants with textured hair (REUG-T, n = 9) yielded a near-identical pattern (15 of 18 comparisons). Signal‑to‑noise ratio at Fz for frequent stimuli increased from sitting to standing (F = 10.33, p = 0.002, adjusted p = 0.036). Behavioral performance was similar across groups. This study provides the first evidence that equitable ERP data quality is achievable across racial groups during active MoBI by combining inclusive hardware with culturally sensitive protocols. These findings confirm that the technological incompatibility underlying historical underrepresentation is surmountable when paired with culturally sensitive protocols, enabling more inclusive and generalizable cognitive neuroscience.

## Author summary

Electroencephalography (EEG) is a vital tool for monitoring brain health, yet standard sensors often fail to record reliable data from individuals with coarse or curly hair. This technological limitation has led to the systematic exclusion of Black participants and others with textured hair from neuroscientific research. In this study, we investigated whether utilizing a specific dry-brush electrode system, paired with culturally sensitive research protocols, could bridge this gap. We compared brain data quality between older adults from racially underrepresented groups and matched White participants during a cognitive task performed while sitting and standing. We found that this inclusive approach produced data of statistically equivalent quality across racial groups, even during the more physically demanding standing condition. Also, total data loss was comparable between White (8.29%) and underrepresented participants (9.88%). This finding is critical because it demonstrates that the historical lack of diversity in EEG research is a solvable problem. By validating that existing inclusive hardware works effectively in dynamic settings, we provide a roadmap for researchers to conduct more equitable studies, ensuring that future brain health innovations benefit all populations equally.

## Introduction

Electroencephalography (EEG) is a widely used method in cognitive neuroscience for understanding both typical and atypical brain function. Compared to other neurophysiological techniques, EEG possesses several unique strengths, including its ability to measure neural activity with unparalleled temporal resolution [1,2]. Furthermore, EEG is noninvasive, highly tolerable, and cost-effective, which makes it exceptionally well-suited for a wide range of clinical applications and lifespan developmental research [1]. A particularly powerful technique derived from EEG is the analysis of event-related potentials (ERPs). ERPs are small voltage fluctuations in the ongoing EEG that are time-locked to the presentation of a specific sensory, cognitive, or motor event [2]. This method allows researchers to isolate and examine the brain’s phased response to discrete stimuli with millisecond precision, making the ERP technique ideal for elucidating the temporal sequence of information processing in the brain.

Despite its broad utility, the generalizability of EEG findings has been significantly compromised by the historical and systematic exclusion of participants from racially and ethnically underrepresented groups (REUG) [1,3,4]. A systematic review of 700 articles highlights the scale of this issue, finding that EEG samples consist predominantly of White participants (78%) from North America and Western Europe, while Blacks, Hispanic/Latino and Asian participants are notably underrepresented [5].

This widespread exclusion is driven by multifaceted systemic issues. For many underrepresented groups, including Hispanic and Asian populations, barriers include a well-founded distrust in medical research stemming from historical exploitation [6,7], linguistic and cultural barriers, and limited access to research opportunities, often due to geographic or socioeconomic factors [6]. For Black individuals, these systemic issues are compounded by a primary technological barrier. Standard EEG caps and sensors have not been designed for these hair textures. [3] Common hairstyles in the Black community such as braids, cornrows, or locs can impede the close scalp adherence required to record reliable and valid EEG data [8,9]. This technological incompatibility has a direct, two-fold impact. First, EEG research may lead to proactive exclusion, where labs may deem these hairstyles as exclusionary due to anticipated poor signal quality [3,4]. Second, even when included, participants with coarse or curly hair may face significant data retention issues, as the poor scalp-electrode connection often results in high impedances and a poor signal-to-noise ratio, rendering the collected data unusable for statistical analysis [10,11].

However, these barriers to inclusion and high-quality data acquisition are not merely technical, but are also deeply embedded in sociocultural factors [3]. The EEG procedure requires physical contact with a person’s hair, which, for Black individuals, intersects with a complex history of trauma and cultural identity [12]. Historically, Black hair has been a target of anti-Black racism and devaluation, second only to skin color as a visible stigma [13,14]. As a result, hair is a crucial component of Black identity and can be an act of resistance [15,16]. This profound personal and cultural significance can lead to a considerable aversion to having one’s hair touched, especially by non-Black researchers [17]. This combination of technological incompatibility, which systematically yields poorer data quality, and sociocultural insensitivity creates a formidable barrier to both participation and high-quality data acquisition from Black individuals [3].

In response to these challenges, the field has begun to develop and validate more inclusive EEG technologies through hardware redesign, novel electrode concepts, and cultural-technical fusion. Pioneering work by Etienne *et al.* [18] demonstrated that combining a culturally-informed hair preparation technique (cornrowing) with a novel clip-based ‘Sevo’ electrode could achieve a greater than 10-fold reduction in impedance on coarse, curly hair in African-American adult participants. Mlandu *et al.* [4] tested a modified high-density infant net with taller sensor pedestals in a South African cohort of infants with curly or tightly coiled hair, showing significantly improved data quality during a resting-state paradigm. Another study developed a ‘stick-and-play’ bioadhesive hairlike electrode that attaches directly to the scalp, demonstrating its ability to enable high-fidelity EEG recordings over 24 hours in participants with dense hair [19]. While these technological advances are promising and demonstrate that the data quality gap can be overcome in the populations studied, their validation has been largely confined to simple, passive paradigms such as resting-state or impedance testing in static, seated positions.

Therefore, this study aims to bridge this ecological validity gap by investigating whether equitable data quality can be achieved during more complex and active paradigms. While Mobile Brain/Body Imaging (MoBI) has expanded EEG into dynamic contexts like postural control [20], the assumption of equivalent data quality across diverse racial groups remains untested. Notably, no study has examined data quality across different racial groups during an ERP paradigm requiring a behavioral response, which introduces additional cognitive and movement-related artifacts. Using a MoBI EEG system, this study directly compared data quality between White and REUG participants during both a standing postural task and a cognitive ERP paradigm. We hypothesized that with inclusive methodologies, data quality would be equivalent between groups, thereby ensuring the generalizability of findings from these advanced mobile and cognitive paradigms.

## Materials and methods

### Participants

Participants were recruited from March 2023 to July 2024 through social media campaigns and personal referrals. To qualify, participants had to: (1) be between 18 and 65 years of age; (2) score at least 26 on the Montreal Cognitive Assessment (MoCA); (3) be able to stand independently for at least two minutes; and (4) follow basic instructions in English. Exclusion criteria included: (1) neurological or musculoskeletal disorders impacting balance; (2) reported cognitive decline or a formal diagnosis of cognitive impairment; and (3) uncorrected hearing loss. Seventeen identified as members of a racial or ethnic REUG participants (including nine Black, one Hispanic, six Asian, and one mixed individual). We individually paired each REUG participant with an age- (absolute age difference of 2.76 years) and sex-matched White participant.

Our protocol was designed using best practice guidelines for inclusive EEG research [3,8,21]. To foster cultural sensitivity and rapport, recruitment, scheduling and procedural discussions were led by research team members who shared racial or ethnic backgrounds with the participants: a Black research member spoke with Black participants, and an Asian research member spoke with Asian participants. These detailed discussions included providing flexible scheduling (e.g., to accommodate hair appointments), recommendations to avoid hair products (like oils or gels) that can increase scalp impedance, and outlining procedures for participants with protective hairstyles such as braids, cornrows, or wigs [21]. For instance, participants with braids or cornrows were collaboratively guided on how these styles could be adapted (e.g., ensuring braids left the scalp along the midline exposed) to allow for direct electrode-to-scalp contact [21]. This collaborative planning was directly linked to our *a priori* methodological choice of a low-density, 8-channel system focused on midline channels. We selected this system over a high-density net precisely because it provides the flexibility to place individual electrodes between braids, prevents the significant noise and channel substitution issues often encountered with high-density systems on diverse hair, and avoids the challenges of acquiring signals from peripheral channels where hair is often thickest.

This cross-sectional, comparative study was approved by the Institutional Review Board of the University of Kansas Medical Center (protocol numbers #00149615, #00150444, and #00150307). All participants provided written informed consent.

### Protocol

#### Collection of demographic information.

We collected basic demographic data, including age, sex, race, and educational background, using a questionnaire created in the REDCap platform. The Montreal Cognitive Assessment (MoCA), scored from 0 to 30, was used to screen participants for their cognitive function [22].

#### Auditory oddball task.

Participants completed an auditory novelty oddball task, identical to the one used in our previous research, [23] while either standing or sitting, with the order randomized [24]. The task involved 50 sound stimuli played through a Bluetooth speaker placed in front of the participant. Of these, 40 (80%) were frequent, low-pitched sounds (600 Hz pure tone burst, 500 ms duration, 10 ms rise/fall time); 5 (10%) were rare, high-pitched sounds (1200 Hz pure tone burst, 500 ms duration, 10 ms rise/fall time); [25] and the remaining 5 (10%) were novelty sounds, including inanimate (e.g., music, video games), animate (e.g., cat, bird), or human sounds, with durations ranging from 159 to 399 ms. The interstimulus interval (ISI) was 1900 ms, with a random jitter of ± 200 ms. While the stimuli were presented in a random order, each rare or novelty sound was always preceded by a frequent sound. The total task duration was approximately 120 seconds. Stimuli were generated using E-Prime 3.0 software. Participants were instructed to use the bottom button on a Bluetooth clicker to respond to frequent stimuli, the top button for rare stimuli, and to ignore novelty sounds [26]. Before the main trials, participants completed a brief practice block to ensure the sounds were audible and that they could correctly distinguish the tones, which all participants successfully did. To reduce motor performance variability, participants were instructed to use their dominant hand for responses, with all participants being right-handed. Participants were reminded to pay equal attention to both the balance and cognitive tasks throughout the experiment.

#### Electroencephalography recording and processing.

Participants were fitted with a MoBI EXG net (Mentalab, Munich, Germany) to record EEG signals at a sampling rate of 250 Hz. Our inclusive hardware strategy centered on a low-density, 8-channel system (Fz, FCz, Cz, CPz, Pz, Oz, Fpz, and Fp1) equipped with dry brush electrodes. The Fp1 electrode was included specifically to facilitate the detection and subsequent filtering of ocular artifacts. This system was deliberately chosen over a high-density net with standard flat electrodes to overcome two key barriers to inclusion. First, the flexible prongs of the brush electrodes are designed to gently part diverse hair types and textures, including coarse or curly hair, to establish a reliable scalp connection and address signal-to-noise challenges where hair might otherwise impede contact [27]. Second, the low-density, midline-focused setup was chosen for its compatibility with protective hairstyles like braids or cornrows [3].

A wet reference electrode was placed on the mastoid. During data collection, electrode impedance was consistently maintained below 30 kΩ. Behavioral data (response time, accuracy) were collected concurrently using E-Prime, and the EEG and behavioral data streams were synchronized via the Lab Streaming Layer [28]. All data processing was performed using MATLAB R2023b [29], EEGLab, [30] and ERPLab v11.03 [31] toolboxes. The raw, continuous EEG signals were first filtered between 0.1 and 30 Hz. Following this, the clean_raw_data function was used to apply Artifact Subspace Reconstruction (ASR) to reduce artifacts in the filtered data. [32] Subsequently, the cleaned data were epoched into segments ranging from -200 ms before stimulus onset to 800 ms after. Epochs were automatically rejected if the voltage in any channel exceeded ±100 µV.

#### Sitting and standing conditions.

Participants completed the sitting task in a seated condition, where they relaxed in an armchair, looked straight ahead, and used their dominant hand to respond to a two-minute auditory oddball task. All participants were right-handed. Subsequently, in the standing condition, participants performed the same auditory oddball task while undergoing the quiet standing protocol (condition 1) of the Virtual Reality Sensory Organization Test (VR-SOT). This protocol was delivered using the VR-COMBAT system [33]. Participants stood barefoot on an AMTI force plate with their feet apart. They wore an HTC Vive Pro Eye headset that displayed a 3D virtual room with patterned walls to serve as a gaze focal point. Instructions were to keep their eyes open, arms relaxed at their sides, and hold the response clicker in their right hand. To ensure safety, a spotter remained present at all times, and participants were secured with a safety harness to prevent falls.

### Analyses

#### ERP data quality analysis.

We assessed the ERP data quality by calculating the Standardized Measurement Error (SME) according to the methodology of Luck et al. [34] where lower SME scores indicate higher precision. A glossary of key technical terms is provided in S1 Table.

For the 50% fractional area latency, we estimated the SME using a bootstrapping approach (bSME). This involved a process, repeated 10,000 times separately for sitting and standing data, where a new waveform was created by randomly selecting trials with replacement from each stimulus type (frequent, rare, novelty). The standard deviation of the 10,000 latency values derived from this procedure was used as the bSME [34].

For the P3 mean amplitude at the Pz and Fz sites, we computed the analytic SME (aSME). This was a three-step process: (1) we found the mean amplitude for every single trial within a 250–550 ms post-stimulus window; (2) we calculated the standard deviation of these single-trial amplitudes; and (3) we divided that standard deviation by the square root of the total trial count to get the aSME. Finally, similar linear mixed models were applied to compare the resulting SME values between groups and conditions.

To further substantiate comparability of data quality, we performed equivalence testing on SME for P3 amplitude and latency. Unlike conventional null-hypothesis tests, which can only fail to reject a difference, equivalence testing asks whether the between-group mean difference falls inside a pre-specified equivalence window [−SESOI, + SESOI], where SESOI is the smallest effect size of interest. We used the two one-sided tests (TOST) procedure: two one-sided t-tests are run, one against the lower bound and one against the upper, and the groups are declared statistically equivalent only when both reject at p < 0.05. TOST was applied separately to each stimulus type (frequent, rare, novelty), each postural condition (sitting, standing), and the within-subject posture change (Δ = standing − sitting). SESOI was anchored to published per-trial SME norms for the P3b component in healthy adults (±1.64 µV for amplitude and ±34.62 ms for latency, taken as the midpoints of the reported ranges (1.13–2.15 µV; 33–36 ms) [34]. A difference smaller than these bounds is below the psychometric resolution at which P3 measurements typically differ across published cohorts. Analyses were performed in R (RStudio 2024.12.0) using TOSTER v0.4.2 with α = 0.05.

As a complementary index of data quality, signal-to-noise ratio (SNR) was computed at the Fz and Pz electrode sites using the plus–minus average method [35–37]. For each participant, condition, channel, and stimulus combination, alternating single-trial epochs were inverted via multiplication by −1 and averaged across all trials to yield a noise-only waveform where the phase-locked ERP component cancels. The standard ERP average over the original, non-inverted trials served as the signal-plus-noise waveform. SNR in decibels was subsequently calculated as the log-transformed ratio of the root-mean-square amplitude of the signal waveform to the root-mean-square amplitude of the noise waveform, with both quantities extracted within the 250–550 ms post-stimulus window. Group differences in SNR were evaluated using linear mixed-effects models mirroring the fixed-effects structure (Group, Condition, and Group x Condition interaction).

Bonferroni correction was applied within the SME and SNR data-quality domains separately (18 omnibus comparisons each; adjusted α = 0.05/18 = 0.0028), because SME was the a priori primary endpoint with formal equivalence testing (TOST) while SNR served as a supplementary sensitivity metric. The pre‑specified SME TOSTs were evaluated at α = 0.05 without further adjustment. All analyses were conducted in RStudio (version 2024.12.0) using the TOSTER (version 0.4.2) package, with a significance threshold set at p < 0.05.

#### Univariate analysis for p3 latency and amplitude.

We analyzed the P3 component elicited by frequent, rare, and novel stimuli within a 250–550 ms post-stimulus time window. Based on established topography [38], we calculated the P3b amplitude and latency at the Pz electrode, as it is typically maximal at parietal sites. To assess the P3a, which is more frontally distributed, we extracted its amplitude and latency from the Fz electrode specifically for novel stimuli [38]. For both components, we computed the mean amplitude and the 50% fractional area latency. Linear mixed models (LMMs) were used to compare these P3 amplitudes and latencies. The models included a random intercept for subject to account for the correlation of repeated measures. We examined the fixed effects of group (White vs. REUG), condition (standing vs. sitting), and the group by condition interaction. Bonferroni correction was applied to the 18 omnibus ERP amplitude and latency tests (adjusted α = 0.0028). Analyses were performed in SAS 9.4, with statistical significance set at p < 0.05.

## Results

### Participants

Participant characteristics are summarized in Table 1. The White and REUG participant groups were well-matched on all demographic and clinical variables. There were no significant group differences in age (p = 0.87), MoCA scores (p = 1.00), or years of education (p = 0.29). Additionally, the distribution of sexes was identical between the two groups (p = 1.00).

**Table 1:** Description of demographic and clinical variables.

Variable	White (17)	REUG (N = 17)	p-value
Age	51.47 ± 24.22	51.29 ± 23.90	0.87
Sex (F)	10 (58.8%)	10 (58.8%)	1.00
Education	17.00 ± 2.87	16.71 ± 3.70	0.29
MoCA	28.65 ± 1.06	28.59 ± 0.94	1.00

Variables are described as mean ± standard deviation or frequency (percentage). Group differences for continuous variables were assessed using Paired T-Tests (for Age) or Wilcoxon Signed-Rank Tests (for MoCA and Education, due to non-normal differences), while the difference for the categorical variable (Sex) was assessed using McNemar’s test.

### Behavioral performance

Behavioral performance on the auditory oddball task is summarized in Table 2. For reaction time to frequent stimuli, the analysis revealed no significant main effect of group (F = 0.28, p = 0.61) or condition (F = 0.00, p = 0.99), and no significant group × condition interaction (F = 1.20, p = 0.29). Similarly, for reaction time to rare stimuli, no significant main effects or interactions were found (all p > 0.05).

**Table 2:** Summary of behavioral outcomes during the auditory oddball task.

Stimulus	Group	Sit (Mean ± SD)	Stand (Mean ± SD)	Group (F, p)	Condition (F, p)	Interaction (F, p)
Frequent Reaction Time (ms)	White	421.84 ± 110.80	369.60 ± 185.48	F = 0.28, p = 0.61	F = 0.00, p = 0.99	F = 1.20, p = 0.29
	REUG	382.40 ± 133.70	434.50 ± 120.18			
Rare Reaction Time (ms)	White	532.66 ± 165.61	551.42 ± 273.35	F = 0.21, p = 0.65	F = 0.09, p = 0.77	F = 0.80, p = 0.39
	REUG	552.95 ± 133.79	553.62 ± 195.73			
Frequent Accuracy (/40)	White	37.64 ± 6.86	34.25 ± 11.43	F = 0.01, p = 0.94	F = 0.00, p = 0.95	F = 1.40, p = 0.26
	REUG	34.29 ± 14.68	38.09 ± 3.83			
Rare Accuracy (/5)	White	4.25 ± 1.54	3.92 ± 1.16	F = 1.86, p = 0.19	F = 0.35, p = 0.56	F = 0.08, p = 0.78
	REUG	4.67 ± 0.52	4.55 ± 0.93			

Analysis of accuracy for frequent stimuli also yielded no significant main effects of group (F = 0.01, p = 0.94) or condition (F = 0.00, p = 0.95), nor was there a significant interaction (F = 1.40, p = 0.26). For rare stimuli, accuracy was also comparable across groups and conditions, with no significant effects observed (all p > 0.05).

### ERP data quality analysis

Each group had a total of 850 epochs (17 participants × 50 epochs) per condition. Analysis of total epoch rejection revealed a significant effect of condition (F = 11.32, p = 0.002), with substantially higher data loss during standing compared to sitting. Data loss was minimal during the sitting condition in both groups (White: 0.71% [0.38 ± 0.81]; REUG: 0.12% [0.06 ± 0.24]) but increased markedly during standing (White: 15.88% [8.44 ± 15.40]; REUG: 19.65% [9.28 ± 14.82]). There were no significant group differences in total data loss, with White participants losing 8.29% (141/1700 trials) and REUG participants losing 9.88% (168/1700 trials) of epochs (F = 0.01, p = 0.91), and no significant group × condition interactions (F = 0.05, p = 0.82).

**Fig 1:**
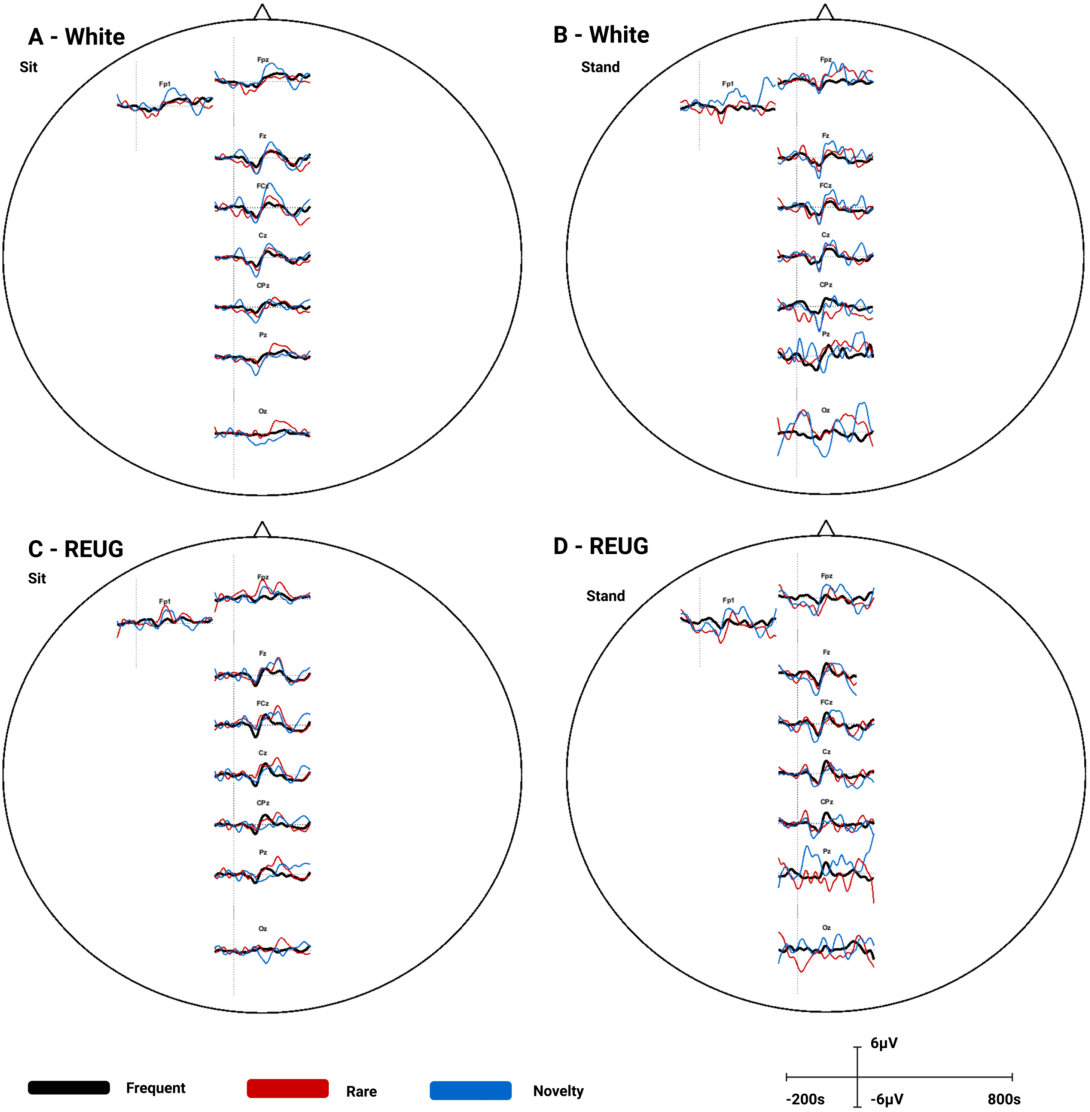
Grand-averaged event-related potential (ERP) waveforms at midline electrode sites. (A, B) White participants during Sitting (A) and Standing (B). (C, D) Racially and Ethnically Underrepresented Group (REUG) participants during Sitting (C) and Standing (D). Black lines represent responses to Frequent stimuli, red lines to Rare stimuli, and blue lines to Novelty stimuli. Negative voltage is plotted downwards.

**Fig 2:**
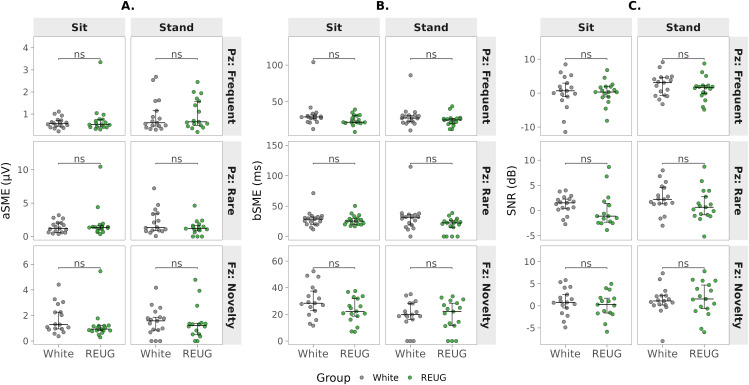
Electrophysiological data quality benchmarks across posture conditions for White and REUG cohorts. (A) Standardized Measurement Error for P3 amplitude (aSME); (B) Bootstrapped Standardized Measurement Error for 50% fractional area latency (bSME); (C) Signal‑to‑Noise Ratio (SNR) quantified using the plus–minus average method within the 250–550 ms post‑stimulus window. No significant main effect of Group was found for any metric across stimulus blocks (Frequent, Rare, Novelty) and postural states (Sit, Stand), and equivalence testing on SME confirmed that data quality was comparable between groups.

The primary outcome of the study, ERP data quality assessed by Standardized Measurement Error (SME), is presented in Table 3 and Fig 1. The analysis revealed no significant main effects of group on any SME measure (Fig 2A and 2B; all p > 0.05) and no significant group-by-condition interactions (all p > 0.05). For the main effect of condition, the SME for novelty P3 latency was nominally lower in the standing condition than in the sitting condition (F = 10.08, p = 0.003); however, this effect did not survive family-wise Bonferroni correction within the SME family (adjusted p = 0.054; S2 Table). No other main effects of condition were statistically significant (all other p > 0.05).

**Table 3:** Summary of standardized measurement error for P3 latency and amplitude.

Stimulus	Variable	Group	Sit (Mean ± SD)	Stand (Mean ± SD)	Group (F, p)	Condition (F, p)	Interaction (F, p)
Frequent	P3 Amplitude (µV)	White	0.60 ± 0.24	0.93 ± 0.75	F = 0.49, p = 0.49	F = 3.82, p = 0.06	F = 0.04, p = 0.83
		REUG	0.74 ± 0.70	1.01 ± 0.70			
	P3 Latency (ms)	White	32.53 ± 19.49	30.05 ± 15.81	F = 2.27, p = 0.14	F = 0.54, p = 0.47	F = 0.48, p = 0.49
		REUG	24.76 ± 7.27	24.70 ± 8.14			
Rare	P3 Amplitude (µV)	White	1.44 ± 0.88	2.19 ± 1.92	F = 0.15, p = 0.70	F = 0.03, p = 0.86	F = 2.65, p = 0.11
		REUG	1.95 ± 2.35	1.36 ± 1.13			
	P3 Latency (ms)	White	28.73 ± 13.04	31.17 ± 24.13	F = 2.24, p = 0.14	F = 1.19, p = 0.28	F = 4.22, p = 0.05
		REUG	26.84 ± 8.63	18.91 ± 12.23			
Novelty	P3 Amplitude (µV)	White	1.73 ± 1.13	1.49 ± 1.11	F = 1.05, p = 0.31	F = 0.00, p = 0.97	F = 0.61, p = 0.44
		REUG	1.21 ± 1.14	1.42 ± 1.34			
	P3 Latency (ms)	White	30.21 ± 12.25	19.19 ± 10.94	F = 1.41, p = 0.24	**F = 10.08, p = 0.003**	F = 2.22, p = 0.15
		REUG	23.12 ± 9.61	19.14 ± 11.60			

### ERP data quality equivalence

Equivalence testing results confirmed that ERP data quality was comparable across groups (Table 4). All P3 amplitude and latency SME comparisons, across stimulus types (frequent, rare, and novelty) and postural conditions (sitting, standing, and change in posture), fell within the specified SESOI bounds (±1.64 µV for amplitude; ± 34.6 ms for latency). For all tests, both one-sided p-values were below the 0.05 threshold (pTOST < 0.05).

**Table 4:** Equivalence testing of data quality across groups and postural conditions.

Stimulus	Channel (SME)	Test Type	Mean ± SD (White)	Mean ± SD (REUG)	Mean Difference	90% CI	TOST p1/ p2	SESOI	Equivalence Decision
Amplitude	Frequent	Δ Posture	0.33 ± 0.72	0.27 ± 1.06	0.07	-0.46, 0.59	3.61e-6/ 1.14e-5	1.64	**Equivalent**
		Sitting	0.60 ± 0.24	0.74 ± 0.70	-0.14	-0.45, 0.17	3.65e-8/ 2.33e-09	1.64	**Equivalent**
		Standing	0.93 ± 0.75	1.01 ± 0.70	-0.07	-0.50, 0.35	2.34e-7/ 4.21e-08	1.64	**Equivalent**
	Rare	Δ Posture	0.74 ± 2.07	-0.70 ± 2.71	1.43	-0.02, 2.88	5.99e-4/ 4.05e-1	1.64	Not equivalent
		Sitting	1.44 ± 0.88	1.95 ± 2.35	-0.51	-1.56, 0.54	3.96e-2/ 1.02e-3	1.64	**Equivalent**
		Standing	2.19 ± 1.92	1.36 ± 1.13	0.84	-0.12, 1.79	8.39e-5/ 8.08e-2	1.64	Not equivalent
	Novelty	Δ Posture	-0.24 ± 1.68	0.19 ± 1.97	-0.43	-1.51, 0.65	3.36e-2/ 1.46e-3	1.64	**Equivalent**
		Sitting	1.73 ± 1.13	1.21 ± 1.14	0.52	-0.14, 1.18	2.12e-6/ 3.70e-3	1.64	**Equivalent**
		Standing	1.49 ± 1.11	1.42 ± 1.34	0.07	-0.66, 0.80	2.02e-4/ 4.85e-4	1.64	**Equivalent**
Latency	Frequent	Δ Posture	-2.48 ± 10.21	-0.06 ± 10.06	-2.42	-8.31, 3.47	7.21e-11/ 2.38e-12	34.62	**Equivalent**
		Sitting	32.53 ± 19.49	24.76 ± 7.27	7.77	-0.92, 16.46	2.36e-8/ 1.56e-5	34.62	**Equivalent**
		Standing	30.05 ± 15.81	24.70 ± 8.14	5.35	-2.03, 12.73	1.10e-9/ 2.60e-7	34.62	**Equivalent**
	Rare	Δ Posture	2.43 ± 14.95	-7.93 ± 14.44	10.36	1.82, 18.90	1.73e-10/ 1.72e-05	34.62	**Equivalent**
		Sitting	28.73 ± 13.04	26.84 ± 8.63	1.89	-4.56, 8.35	1.20e-10/ 1.21e-09	34.62	**Equivalent**
		Standing	31.17 ± 24.13	18.91 ± 12.23	12.26	1.02, 23.49	1.18e-7/ 1.17e-3	34.62	**Equivalent**
	Novelty	Δ Posture	-11.02 ± 16.38	-3.98 ± 10.54	-7.04	-15.09, 1.00	1.56e-06/ 8.85e-10	34.62	**Equivalent**
		Sitting	30.21 ± 12.25	23.12 ± 9.61	7.09	0.68, 13.50	1.93e-12/ 1.94e-08	34.62	**Equivalent**
		Standing	19.19 ± 10.94	19.14 ± 11.60	0.05	-6.50, 6.60	1.58e-10/ 1.68e-10	34.62	**Equivalent**

**Fig 3:**
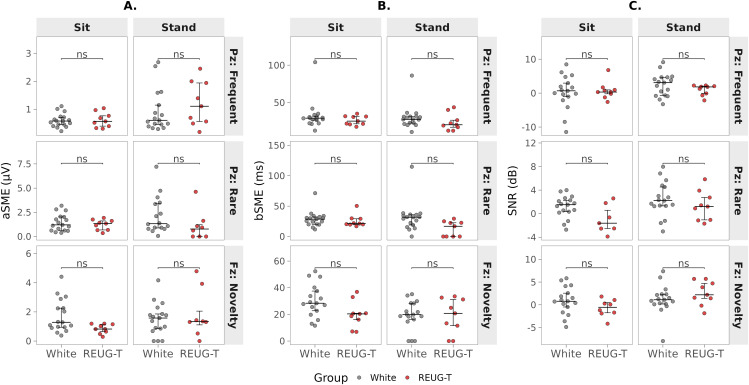
Subgroup sensitivity analysis of ERP data quality in White versus Textured-Hair REUG participants. Individual-level Standardized Measurement Error (SME) for (A) P3 amplitude (aSME, µV), (B) P3 latency (bSME, ms), and (C) signal-to-noise ratio (SNR, dB), stratified by stimulus type (rows: Pz: Frequent, Pz: Rare, Fz: Novelty) and postural condition (columns: Sit, Stand).

In the sensitivity analysis restricted to REUG participants with self‑reported textured hair (REUG‑T, n = 9; Fig 3 and S3 Table), TOST confirmed equivalence for all 9 SME latency comparisons and for 6 of the 9 SME amplitude comparisons (15 of 18 total). The three amplitude comparisons that did not meet the equivalence criterion were those for the Rare stimulus at Pz, across all three postural contrasts (Sit, Stand, and Δ‑posture).

### Signal‑to‑noise ratio

SNR values at Pz and Fz were comparable between groups across all stimulus types and postural conditions (S4 Table and Fig 2C). Linear mixed‑effects models revealed no significant main effects of Group (raw p values range 0.101–0.866; all Bonferroni‑adjusted p = 1.000) and no Group × Condition interactions (p values range 0.409–0.859; all adjusted p = 1.000). A single main effect of Condition survived family‑wise Bonferroni correction within the SNR family: SNR at Fz for frequent stimuli increased from sitting to standing (F = 10.33, p = 0.002, adjusted p = 0.036). No other Condition effects approached significance (all p > 0.05).

### P3 latency and amplitude

The key findings for each stimulus type are detailed below, with all descriptive and inferential statistics presented in Table 5. For frequent stimuli, no significant main effects or interactions were found for either P3 amplitude or P3 latency (all p > 0.05).

**Table 5:** P3 latency and amplitude during sitting and standing conditions.

Stimulus	Variable	Group	Sit (Mean ± SD)	Stand (Mean ± SD)	Group (F, p)	Condition (F, p)	Interaction (F, p)
Frequent	P3 Amplitude (µV)	White	0.45 ± 0.79	0.53 ± 2.32	F = 0.32, p = 0.57	F = 0.00, p = 0.99	F = 0.11, p = 0.74
		REUG	0.32 ± 0.70	0.22 ± 1.02			
	P3 Latency (ms)	White	388.94 ± 38.33	384.47 ± 37.76	F = 0.30, p = 0.59	F = 0.04, p = 0.84	F = 0.67, p = 0.42
		REUG	377.65 ± 29.33	385.18 ± 35.04			
Rare	P3 Amplitude (µV)	White	0.70 ± 1.73	1.53 ± 3.98	F = 2.23, p = 0.15	F = 1.48, p = 0.23	**F = 5.32, p = 0.03**
		REUG	1.32 ± 3.33	-1.35 ± 3.02			
	P3 Latency (ms)	White	390.12 ± 41.45	405.00 ± 34.49	F = 0.37, p = 0.55	F = 0.02, p = 0.90	F = 3.17, p = 0.08
		REUG	400.71 ± 41.36	383.50 ± 26.04			
Novelty	P3 Amplitude (µV)	White	0.68 ± 1.49	1.19 ± 2.07	F = 0.00, p = 0.99	F = 0.07, p = 0.79	F = 2.79, p = 0.10
		REUG	1.28 ± 1.64	0.57 ± 1.44			
	P3 Latency (ms)	White	376.71 ± 38.92	383.06 ± 28.34	F = 4.10, p = 0.05	F = 0.01, p = 0.92	F = 0.43, p = 0.52
		REUG	399.06 ± 41.44	394.50 ± 24.43			

In response to rare stimuli, a nominally significant Group × Condition interaction was observed for P3 amplitude (F = 5.32, p = 0.03); however, this effect did not survive family-wise Bonferroni correction within the P3 family (adjusted p = 0.540 across 18 tests; S5 Table). No significant interaction was observed for the P3 latency (F = 3.17, p = 0.08).

For novelty stimuli, no significant main effects or interactions were found for either P3 amplitude or P3 latency (all p > 0.05).

## Discussion

The primary aim of this study was to determine whether MOBI through an EEG paradigm could produce equitable data quality between White and REUG adults during a cognitive task in sitting and standing. Our principal finding demonstrates that data quality of the ERP, as measured by SME, was comparable across racial groups. We found no significant main effects of group for any SME metric of P3 amplitude or latency. Furthermore, the comparability of data quality held true in both sitting and standing conditions, as shown by the absence of any significant interactions. Complementary equivalence testing further confirmed that data quality metrics were statistically equivalent between groups in 16 of 18 stimulus × postural cells, reinforcing the robustness of these findings. Taken together, these findings provide strong evidence that MOBI EEG technologies, when applied with inclusive methods, can acquire high-quality, comparable brain activity recordings from diverse racial groups, even during cognitively demanding, postural control tasks. This study, therefore, contributes meaningfully to inclusive research in cognitive neuroscience by demonstrating that equivalence in EEG data quality across diverse populations is achievable through intentional design.

Our finding of comparable data quality across groups, even during the more challenging standing condition, is particularly significant. Research demonstrates that as paradigms become more active and ecologically valid, concerns about movement artifacts and data quality intensify [39]. Yet, we did not observe a systemic degradation in data quality in our standing condition, and in one case (i.e., SNR for Frequent stimuli) even found an improvement in data quality. The high precision of our data confirms that these concerns can be successfully mitigated. Our standardized measurement error (SME) for P3 amplitude (0.56–2.59 µV) aligns with established benchmarks from standard laboratory paradigms (1.13–2.15 µV; Luck et al. [34]). Notably, our SME for P3 latency (18.91–27.72 ms) demonstrates even greater precision than the reported benchmark of 33–36 ms. [34] Also, the low epoch rejection rates of 8.29% (White) and 9.88% (REUG) in our study compare to benchmarks from other large-scale EEG studies, even those using less demanding, stationary paradigms. A study by Kaiser et al., which investigated data quality in a multi-center trial using standard sitting tasks, reported that their non-Attention-Deficit/Hyperactivity Disorder group alone had an artifact rejection rate of 24–30%, with even higher data loss observed in their clinical population [40]. These results suggest that the primary historical barriers to obtaining high-quality EEG from underrepresented groups, including both technological incompatibility and broader sociocultural factors, are surmountable, and not an inherent limitation of the participants or more demanding paradigms. Therefore, the longstanding underrepresentation documented in the literature [5] may be a problem of methodological approach, not population. As EEG research increasingly adopts advanced computational approaches that depend on clean, artifact‑free signals, the ability to acquire high‑quality raw data across diverse populations becomes foundational to equitable clinical translation [41].

Achieving this equivalence in EEG data quality was not a passive outcome but rather the result of a deliberate, socio-technical approach designed to overcome the multifaceted barriers to inclusion. The use of racially concordant team members for recruitment and communication was a foundational step to build rapport and mitigate the historical distrust and cultural barriers that have systematically excluded underrepresented groups from biomedical research [7,21]. This proactive, participant-centered framework ensured that individuals felt respected and understood, which was essential for both recruitment and the subsequent acquisition of high-quality data [18]. This procedural orientation and the purpose-built dry-brush hardware operated as an existentially codependent pipeline; the cultural protocol resolved relational barriers and ensured compatible hair preparation (such as keeping midline scalp tracts clear), while the hardware resolved the physical obstacle of hair parting required to capture the electrical signal. Hardware improvements alone, however, cannot resolve the deeper systemic distrust and socioeconomic barriers that also drive underrepresentation; those require sustained cultural and relational solutions of the kind embedded in this protocol. Among the procedural steps, the collaborative styling guidance, ensuring that protective styles left the midline scalp exposed, was the single most operationally critical element, as it directly created the physical access window that the midline electrode montage required to function. This strategy demonstrates that overcoming the field’s diversity issue requires addressing not only hardware limitations but also the relational and cultural dynamics of the research process itself.

On the technical side, the hardware itself was crucial. We utilized a dry-electrode system with brush-like prongs, akin to the “fingered electrodes” described by Casson [27], designed to effectively part the hair and ensure firm scalp contact. While previous validation of inclusive hardware has been confined to simple, static paradigms [4,18]. The active standing condition introduces postural and movement artifacts that often degrade EEG signals in real-world settings [42,43]. Our study not only confronted this challenge but demonstrated that high-quality data is attainable. This emphasis on pipeline standardization and validation across heterogeneous populations parallels recent methodological discussions in quantitative biomedical feature analysis [44]. This technical robustness is further highlighted by our targeted sensitivity analysis restricted to the subgroup of participants with self-reported textured hair (REUG-T). Even when focusing on the hair profile that presents the most significant historical barrier to scalp-electrode contact, the inclusive pipeline preserved statistical equivalence in all 9 of 9 latency cells and 6 of 9 amplitude cells. The three cells that did not meet the equivalence criterion were entirely isolated within the low-trial-count amplitude blocks (Rare standing and change-in-posture; Novelty change-in-posture). Because each participant’s individual SME score within these conditions is derived from only 5 trials, it is likely that this trial-count constraint limits the precision of the between-group statistical estimate rather than reflecting a systemic performance failure of the electrode system across textured hair. These subgroup findings suggest that dry-brush hardware designs represent a viable step toward broadening the reach of mobile neuroimaging to populations with diverse hair phenotypes.

The success of our socio-technical approach advances the field on two parallel fronts. First, the strategy directly addresses the urgent need for inclusive hardware. Second, and equally important, the approach demonstrates that inclusivity is achievable even within the demanding MoBI paradigm, a context that typically intensifies the trade-off between ecological validity and data quality [4,18,42]. Future EEG studies can adopt similar socio-technical frameworks to ensure signal quality and interpretability across diverse populations, improving the generalizability of findings. Furthermore, by demonstrating equivalence in EEG data quality, this study supports the inclusion of diverse participants in clinical trials without compromising data integrity, which is crucial for developing treatments that are effective across populations.

Beyond the primary focus on data quality, we also examined behavioral and ERP outcomes. No group differences were found in behavioral performance, confirming equivalent task execution. In the uncorrected P3 analysis, a nominally significant group-by-condition interaction emerged for amplitude to rare stimuli. After family‑wise Bonferroni correction within the P3 inferential family, however, this effect was no longer significant. The interaction therefore most likely reflects sample‑specific variability rather than a robust divergence in cognitive strategy, and any interpretation should await replication in larger cohorts.

### Limitations

Several limitations of this study should be considered when interpreting its findings. First, our study was conducted in a controlled laboratory environment. Although the standing condition introduced greater postural and cognitive demands, it does not represent the full complexity of real-world, over-ground mobility. Furthermore, the findings are based on a single auditory oddball paradigm and may not generalize to other cognitive tasks or clinical EEG applications. The ecological validity of our findings for fully naturalistic MoBI applications, such as walking in a crowded space, remains to be established. Future work should test this socio-technical model in truly unconstrained environments to determine the outer limits of its effectiveness.

Second, our reliance on a low-density, 8-channel EEG montage restricts spatial resolution and limits the evaluation of nuanced ERP topography or cortical source localization. However, this midline configuration was an intentional methodological choice designed to maximize inclusivity. Midline channels are uniquely accessible across diverse hair profiles, allowing electrodes to be placed efficiently within protective hairstyles like braids or cornrows to achieve direct scalp contact. Because the P3 component is functionally maximal and robustly quantified along the midline, this setup preserved the integrity of our primary cognitive endpoints while minimizing participant burden. Future work should evaluate emerging inclusive sensor designs that expand channel counts without compromising equitable data acquisition.

Also, our participant pool was restricted to high-functioning, cognitively healthy adults. It is unclear whether these methods would be equally effective and tolerable in clinical populations with significant cognitive or motor impairments, where cooperation may be more challenging and movement artifacts more pronounced. Validating this approach in populations with Mild Cognitive Impairment, Alzheimer’s disease, or Parkinson’s disease is a critical next step. Finally, while the subgroup analysis represents a major methodological strength by demonstrating that signal equivalence is preserved even across the hair profiles historically most marginalized by standard EEG hardware, we must still caution against overgeneralization. A cohort of nine individuals cannot capture the immense structural diversity within coarse, curly, or tightly coiled hair phenotypes. Future research utilizing larger, intentionally powered, and demographically broader cohorts is necessary to validate these pipeline metrics at scale and evaluate the intersecting nuances unique to specific racial, ethnic, and cultural subgroups.

## Conclusion

This study provides the first empirical evidence that equitable EEG data quality can be achieved across racial groups during an active MoBI paradigm. By adopting a deliberate socio-technical approach, combining culturally sensitive protocols with purpose-built, dry-electrode hardware, we successfully bridged the data-quality gap that has long hindered the inclusive application of EEG research. These findings lay the groundwork for broader adoption of inclusive EEG methodologies in both research and clinical settings.

## Supporting information

S1 Table Glossary of key technical terms. (DOCX)

S2 Table Bonferroni‑corrected F‑statistics and p‑values for the SME family. (DOCX)

S3 Table Equivalence testing (TOST) results for the textured‑hair subgroup (REUG‑T). (DOCX)

S4 Table Signal‑to‑noise ratio (SNR) results by group and condition. (DOCX)

S5 Table Bonferroni‑corrected F‑statistics and p‑values for the P3 family. (DOCX)

S1 Dataset Minimal underlying data: This file contains the de-identified demographic, behavioral, and EEG data (including SME, amplitude, latency, and artifact rejection rates) used for all statistical analyses presented in this study. (XLSX)
