# Supplementary material for: Inclusive mobile brain-body imaging achieves equivalent EEG data quality across racial groups
Source: PLOS Digit Health. 2026 Aug 7;5(8):e0001638. doi: 10.1371/journal.pdig.0001638 (PMC13450788; doi:10.1371/journal.pdig.0001638)
Supplement: Supplementary file 1 [file pdig.0001638.s001.docx]

**S1 Table. Glossary of key technical terms.**

| **Term** | **Definition** |
| --- | --- |
| EEG | Electroencephalography; a non-invasive method that records electrical activity of the brain via electrodes placed on the scalp. |
| ERP | Event-related potential; a small voltage fluctuation in the ongoing EEG that is time-locked to a specific sensory, cognitive, or motor event. |
| MoBI | Mobile Brain/Body Imaging; an approach that combines EEG recording with simultaneous measurement of body movements to study brain activity in active, ecologically valid settings. |
| P3 (P300) | A positive-going ERP component peaking approximately 300–600 ms post-stimulus, associated with attentional resource allocation and cognitive processing. |
| P3b | A parietal-maximal subcomponent of the P3 elicited by task-relevant or target stimuli, reflecting context updating and attentional resource allocation. |
| P3a | A frontocentral-maximal subcomponent of the P3 elicited by novel or unexpected stimuli, reflecting involuntary orienting of attention. |
| Pz | A midline parietal electrode site on the international 10–20 EEG system, where the P3b component is typically maximal. |
| Fz | A midline frontal electrode site on the international 10–20 EEG system, where the P3a component is typically maximal. |
| SME | Standardized Measurement Error; a metric that quantifies the expected measurement error of an averaged ERP parameter (amplitude or latency). Lower values indicate higher precision. |
| aSME | Analytic SME; computed directly from single-trial amplitude values as the standard deviation divided by the square root of the number of trials. Used for amplitude measures. |
| bSME | Bootstrapped SME; estimated via resampling (10,000 iterations) to quantify measurement error of latency measures, which cannot be derived analytically. |
| 50% fractional area latency | The time point at which 50% of the total area under the ERP waveform has accumulated; a robust measure of component timing. |
| SNR | Signal-to-noise ratio; the ratio of the ERP signal amplitude to the background EEG noise amplitude, expressed in decibels (dB). Higher values indicate cleaner signal. |
| TOST | Two one-sided tests; a statistical procedure used to test for equivalence between two groups by determining whether the difference falls within a pre-specified equivalence bound. |
| SESOI | Smallest effect size of interest; the minimum difference considered practically or theoretically meaningful, used to define equivalence bounds in TOST. |
| REUG | Racially and ethnically underrepresented groups; in this study, participants identifying as Black, Hispanic, Asian, or mixed race. |
| REUG-T | The subset of REUG participants with self-reported textured hair (all Black, n=9). |
| Auditory oddball task | A cognitive paradigm in which participants detect infrequent target stimuli (rare, novelty) interspersed among frequent standard stimuli, used to elicit the P3 component. |
