# Supplementary material for: Inclusive mobile brain-body imaging achieves equivalent EEG data quality across racial groups
Source: PLOS Digit Health. 2026 Aug 7;5(8):e0001638. doi: 10.1371/journal.pdig.0001638 (PMC13450788; doi:10.1371/journal.pdig.0001638)
Supplement: Supplementary file 2 [file pdig.0001638.s002.docx]

**S2 Table. Bonferroni‑corrected F‑statistics and p‑values for the SME**

| **Stimulus** | **Variable** | **Group** | **Sit (Mean ± SD)** | **Stand (Mean ± SD)** | **Group (F, p_adj)** | **Condition (F, p_adj)** | **Interaction (F, p_adj)** |
| --- | --- | --- | --- | --- | --- | --- | --- |
| Frequent | P3 Amplitude (µV) | White | 0.60 ± 0.24 | 0.93 ± 0.75 | F=0.49, p=1.000 | F=3.82, p=1.000 | F=0.04, p=1.000 |
|  |  | REUG | 0.74 ± 0.70 | 1.01 ± 0.70 |  |  |  |
|  | P3 Latency (ms) | White | 27.43 ± 6.92 | 25.83 ± 7.10 | F=1.00, p=1.000 | F=0.25, p=1.000 | F=0.21, p=1.000 |
|  |  | REUG | 24.76 ± 7.27 | 24.70 ± 8.14 |  |  |  |
| Rare | P3 Amplitude (µV) | White | 1.44 ± 0.88 | 2.19 ± 1.92 | F=0.15, p=1.000 | F=0.03, p=1.000 | F=2.65, p=1.000 |
|  |  | REUG | 1.95 ± 2.35 | 1.36 ± 1.13 |  |  |  |
|  | P3 Latency (ms) | White | 25.69 ± 7.24 | 26.01 ± 10.88 | F=1.29, p=1.000 | F=3.04, p=1.000 | F=3.58, p=1.000 |
|  |  | REUG | 26.84 ± 8.63 | 18.91 ± 12.23 |  |  |  |
| Novelty | P3 Amplitude (µV) | White | 1.73 ± 1.13 | 1.49 ± 1.11 | F=1.05, p=1.000 | F=0.00, p=1.000 | F=0.61, p=1.000 |
|  |  | REUG | 1.21 ± 1.14 | 1.42 ± 1.34 |  |  |  |
|  | P3 Latency (ms) | White | 27.72 ± 11.77 | 18.91 ± 11.02 | F=0.52, p=1.000 | F=7.87, p=0.108 | F=1.12, p=1.000 |
|  |  | REUG | 23.12 ± 9.61 | 19.14 ± 11.60 |  |  |  |
