# Supplementary material for: Inclusive mobile brain-body imaging achieves equivalent EEG data quality across racial groups
Source: PLOS Digit Health. 2026 Aug 7;5(8):e0001638. doi: 10.1371/journal.pdig.0001638 (PMC13450788; doi:10.1371/journal.pdig.0001638)
Supplement: Supplementary file 3 [file pdig.0001638.s003.docx]

**S3 Table. Signal‑to‑noise ratio (SNR) results by group and condition.**

| **Stimulus** | **Variable** | **Group** | **Sit (Mean ± SD)** | **Stand (Mean ± SD)** | **Group (F, p)** | **Condition (F, p)** | **Interaction (F, p)** |
| --- | --- | --- | --- | --- | --- | --- | --- |
| Frequent | P3 Amplitude (µV) | White | 2.79 ± 3.78 | 6.03 ± 3.12 | F=2.36, p=0.13 | **F=10.33, p=0.00** | F=0.69, p=0.41 |
|  |  | REUG | 2.23 ± 3.08 | 4.14 ± 3.18 |  |  |  |
| Rare | P3 Amplitude (µV) | White | 1.41 ± 2.40 | 0.59 ± 2.14 | F=0.03, p=0.87 | F=0.51, p=0.48 | F=0.36, p=0.55 |
|  |  | REUG | 1.17 ± 2.98 | 1.08 ± 2.89 |  |  |  |
| Novelty | P3 Amplitude (µV) | White | 0.93 ± 2.99 | 1.20 ± 3.21 | F=0.04, p=0.84 | F=1.23, p=0.28 | F=0.59, p=0.45 |
|  |  | REUG | 0.15 ± 2.98 | 1.63 ± 3.89 |  |  |  |
| Frequent | P3 Latency (ms) | White | 0.28 ± 5.00 | 2.43 ± 3.37 | F=0.32, p=0.58 | F=3.70, p=0.06 | F=0.37, p=0.55 |
|  |  | REUG | 0.23 ± 3.36 | 1.34 ± 3.37 |  |  |  |
| Rare | P3 Latency (ms) | White | 1.16 ± 1.86 | 2.43 ± 2.91 | F=2.77, p=0.10 | F=2.52, p=0.12 | F=0.03, p=0.86 |
|  |  | REUG | 0.08 ± 3.56 | 1.10 ± 3.21 |  |  |  |
| Novelty | P3 Latency (ms) | White | -0.79 ± 2.34 | -0.21 ± 3.36 | F=0.19, p=0.66 | F=0.19, p=0.66 | F=0.15, p=0.70 |
|  |  | REUG | -0.21 ± 2.29 | -0.17 ± 3.11 |  |  |  |
