# Supplementary material for: Inclusive mobile brain-body imaging achieves equivalent EEG data quality across racial groups
Source: PLOS Digit Health. 2026 Aug 7;5(8):e0001638. doi: 10.1371/journal.pdig.0001638 (PMC13450788; doi:10.1371/journal.pdig.0001638)
Supplement: Supplementary file 4 [file pdig.0001638.s004.docx]

**Table S4. P3 Event-Related Potential (ERP) Amplitude and Latency Outcomes with Composite Bonferroni Corrections**

| **Stimulus** | **Variable** | **Group** | **Sit (Mean ± SD)** | **Stand (Mean ± SD)** | **Group (F, p_adj)** | **Condition (F, p_adj)** | **Interaction (F, p_adj)** |
| --- | --- | --- | --- | --- | --- | --- | --- |
| Frequent | P3 Amplitude (µV) | White | 0.45 ± 0.79 | 0.53 ± 2.32 | F=0.32, p=1.000 | F=0.00, p=1.000 | F=0.11, p=1.000 |
|  |  | REUG | 0.32 ± 0.70 | 0.22 ± 1.02 |  |  |  |
|  | P3 Latency (ms) | White | 388.94 ± 38.33 | 384.47 ± 37.76 | F=0.30, p=1.000 | F=0.04, p=1.000 | F=0.67, p=1.000 |
|  |  | REUG | 377.65 ± 29.33 | 385.18 ± 35.04 |  |  |  |
| Rare | P3 Amplitude (µV) | White | 0.70 ± 1.73 | 1.53 ± 3.98 | F=2.23, p=1.000 | F=1.48, p=1.000 | F=5.32, p=1.000 |
|  |  | REUG | 1.32 ± 3.33 | -1.35 ± 3.02 |  |  |  |
|  | P3 Latency (ms) | White | 390.12 ± 41.45 | 405.00 ± 34.49 | F=0.37, p=1.000 | F=0.02, p=1.000 | F=3.17, p=1.000 |
|  |  | REUG | 400.71 ± 41.36 | 383.50 ± 26.04 |  |  |  |
| Novelty | P3 Amplitude (µV) | White | 0.68 ± 1.49 | 1.19 ± 2.07 | F=0.00, p=1.000 | F=0.07, p=1.000 | F=2.79, p=1.000 |
|  |  | REUG | 1.28 ± 1.64 | 0.57 ± 1.44 |  |  |  |
|  | P3 Latency (ms) | White | 376.71 ± 38.92 | 383.06 ± 28.34 | F=4.10, p=1.000 | F=0.01, p=1.000 | F=0.43, p=1.000 |
|  |  | REUG | 399.06 ± 41.44 | 394.50 ± 24.43 |  |  |  |
