# Supplementary material for: Inclusive mobile brain-body imaging achieves equivalent EEG data quality across racial groups
Source: PLOS Digit Health. 2026 Aug 7;5(8):e0001638. doi: 10.1371/journal.pdig.0001638 (PMC13450788; doi:10.1371/journal.pdig.0001638)
Supplement: Supplementary file 5 [file pdig.0001638.s005.docx]

**S5 Table. Equivalence testing (TOST) results for the textured‑hair subgroup (REUG‑T).**

| **Outcome** | **Channel (SME)** | **Test Type** | **Mean ± SD (White)** | **Mean ± SD (REUG)** | **Mean Difference** | **90% CI** | **TOST p1 / p2** | **SESOI** | **Equivalence Decision** |
| --- | --- | --- | --- | --- | --- | --- | --- | --- | --- |
| Amplitude (µV) | Frequent | Δ Posture | 0.33 ± 0.72 | 0.58 ± 0.78 | -0.25 | -0.80, 0.30 | 2.31e-4 / 1.04e-5 | 1.64 | **Equivalent** |
|  |  | Sitting | 0.60 ± 0.24 | 0.62 ± 0.27 | -0.02 | -0.21, 0.17 | 7.82e-11 / 5.54e-11 | 1.64 | **Equivalent** |
|  |  | Standing | 0.93 ± 0.75 | 1.20 ± 0.79 | -0.27 | -0.83, 0.29 | 3.04e-4 / 1.06e-5 | 1.64 | **Equivalent** |
|  | Rare | Δ Posture | 0.74 ± 2.07 | -0.21 ± 1.51 | 0.94 | -0.34, 2.23 | 1.29e-3 / 1.80e-1 | 1.64 | Not equivalent |
|  |  | Sitting | 1.44 ± 0.88 | 1.21 ± 0.54 | 0.23 | -0.25, 0.71 | 3.66e-7 / 2.02e-5 | 1.64 | **Equivalent** |
|  |  | Standing | 2.19 ± 1.92 | 1.11 ± 1.54 | 1.08 | -0.18, 2.34 | 7.72e-4 / 2.26e-1 | 1.64 | Not equivalent |
|  | Novelty | Δ Posture | -0.24 ± 1.68 | 0.98 ± 1.71 | -1.22 | -2.50, 0.07 | 2.85e-1 / 8.20e-4 | 1.64 | **Not equivalent** |
|  |  | Sitting | 1.73 ± 1.13 | 0.85 ± 0.32 | 0.88 | 0.37, 1.39 | 1.89e-8 / 9.10e-3 | 1.64 | **Equivalent** |
|  |  | Standing | 1.49 ± 1.11 | 1.83 ± 1.65 | -0.34 | -1.50, 0.83 | 3.52e-2 / 5.85e-3 | 1.64 | **Equivalent** |
| Latency (ms) | Frequent | Δ Posture | -2.48 ± 10.21 | -2.45 ± 11.18 | -0.04 | -7.87, 7.80 | 6.14e-7 / 5.99e-7 | 34.62 | **Equivalent** |
|  |  | Sitting | 32.53 ± 19.49 | 26.20 ± 6.14 | 6.34 | -2.52, 15.20 | 4.43e-8 / 9.39e-6 | 34.62 | **Equivalent** |
|  |  | Standing | 30.05 ± 15.81 | 23.75 ± 11.25 | 6.30 | -2.92, 15.52 | 7.15e-8 / 1.41e-5 | 34.62 | **Equivalent** |
|  | Rare | Δ Posture | 2.43 ± 14.95 | -12.57 ± 15.51 | 15.00 | 3.97, 26.03 | 3.66e-7 / 3.42e-3 | 34.62 | **Equivalent** |
|  |  | Sitting | 28.73 ± 13.04 | 25.46 ± 10.28 | 3.27 | -4.77, 11.31 | 4.35e-8 / 7.39e-7 | 34.62 | **Equivalent** |
|  |  | Standing | 31.17 ± 24.13 | 12.89 ± 12.65 | 18.27 | 5.93, 30.61 | 7.13e-8 / 1.64e-2 | 34.62 | **Equivalent** |
|  | Novelty | Δ Posture | -11.02 ± 16.38 | -1.41 ± 11.70 | -9.61 | -19.18, -0.05 | 9.43e-5 / 3.76e-8 | 34.62 | **Equivalent** |
|  |  | Sitting | 30.21 ± 12.25 | 20.06 ± 10.05 | 10.15 | 2.41, 17.88 | 2.05e-9 / 1.31e-5 | 34.62 | **Equivalent** |
|  |  | Standing | 19.19 ± 10.94 | 18.65 ± 13.20 | 0.53 | -8.52, 9.58 | 4.09e-6 / 5.72e-6 | 34.62 | **Equivalent** |
